# Long-term outcomes of the pentaspline pulsed-field ablation catheter for the treatment of paroxysmal atrial fibrillation: results of the prospective, multicentre FARA-Freedom Study

**DOI:** 10.1093/europace/euae053

**Published:** 2024-02-22

**Authors:** Andreas Metzner, Martin Fiala, Johan Vijgen, Alexandre Ouss, Melanie Gunawardene, Jim Hansen, Josef Kautzner, Boris Schmidt, Mattias Duytschaever, Tobias Reichlin, Yuri Blaauw, Philipp Sommer, Annelies Vanderper, Anitha B Achyutha, Madeline Johnson, Jonathan D Raybuck, Petr Neuzil

**Affiliations:** Klinik für Kardiologie, Universitätsklinikum Hamburg-Eppendorf, Martinistraße 52, Gebäude Ost 70, 20246 Hamburg, Germany; Department of Internal Medicine and Cardiology, University Hospital Brno, Brno, Czech Republic; Division of Electrophysiology, Jessa Ziekenhuis, Hasselt, Belgium; Department of Cardiology, Catharina Ziekenhuis, Eindhoven, The Netherlands; Department of Cardiology and Internal Intensive Care Medicine, Asklepios Klinik St Georg, Hamburg, Germany; Department of Cardiology, Gentofte Hospital, Hellerup, Denmark; Department of Cardiology, Institute for Clinical and Experimental Medicine-IKEM, Prague, Czech Republic; Cardioangiologisches Centrum Bethanien, Academic Teaching Hospital of Goethe University of Frankfurt, Frankfurt, Germany; Department or Cardiology, AZ SINT-Jan AV, Bruges, Belgium; Department of Rhythmology and Cardiac Electrophysiology, Inselspital University Hospital Bern, University of Bern, Bern, Switzerland; Department of Cardiology, University Medical Center, Groningen, The Netherlands; Department of Cardiology and Rhythmology, Hdz Nrw, Bad Oeynhausen, Gemany; AF Solutions, Boston Scientific Corp, St. Paul, MN, USA; AF Solutions, Boston Scientific Corp, St. Paul, MN, USA; AF Solutions, Boston Scientific Corp, St. Paul, MN, USA; AF Solutions, Boston Scientific Corp, St. Paul, MN, USA; Department of Cardiology, Nemocnice Na Homolce Hospital, Prague, Czech Republic

**Keywords:** Pulsed field ablation, Atrial fibrillation, Paroxysmal atrial fibrillation, Pentaspline PFA catheter

## Abstract

**Aims:**

Pulmonary vein isolation (PVI) is a well-established strategy for the treatment of paroxysmal atrial fibrillation (PAF). Despite randomized controlled trials and real-world data showing the promise of pulsed-field ablation (PFA) for this treatment, long-term efficacy and safety data demonstrating single-procedure outcomes off antiarrhythmic drugs remain limited. The aim of the FARA-Freedom Study was to evaluate the long-term efficacy and safety of PFA using the pentaspline catheter for PAF.

**Methods and results:**

FARA-Freedom, a prospective, non-randomized, multicentre study, enrolled patients with PAF undergoing *de novo* PVI with PFA, who were followed for 12 months with weekly transtelephonic monitoring and a 72-h Holter ECG at 6 and 12 months. The primary safety endpoint was a composite of device- or procedure-related serious adverse events out to 7 days post-ablation and PV stenosis or atrioesophageal (AE) fistula out to 12 months. Treatment success is a composite of acute PVI and chronic success, which includes freedom from any documented atrial tachyarrhythmia longer than 30 s, use of antiarrhythmic drugs or cardioversion after a 3-month blanking period, or use of amiodarone or repeat ablation at any time. The study enrolled 179 PAF patients (62 ± 10 years, 39% female) at 13 centres. At the index procedure, all PVs were successfully isolated with the pentaspline PFA catheter. Procedure and left atrial dwell times, with a 20-min waiting period, were 71.9 ± 17.6 and 41.0 ± 13.3 min, respectively. Fluoroscopy time was 11.5 ± 7.4 min. Notably, monitoring compliance was high, with 88.4 and 90.3% with weekly events and 72-h Holter monitors, respectively. Freedom from the composite primary effectiveness endpoint was 66.6%, and 41 patients had atrial tachyarrhythmia recurrence, mostly recurrent atrial fibrillation (31 patients). The composite safety endpoint occurred in two patients (1.1%), one tamponade and one transient ischaemic attack. There was no coronary spasm, PV stenosis, or AE fistula. There were four cases of transient phrenic nerve palsy, but all resolved during the index procedure.

**Conclusion:**

In this prospective, non-randomized, multicentre study, PVI using a pentaspline PFA catheter was effective in treating PAF patients despite rigourous endpoint definitions and high monitoring compliance and demonstrated favourable safety.

**Registration:**

Clinical Trials.gov Identifier: NCT05072964 (sponsor: Boston Scientific Corporation).

## Graphical Abstract

**Graphical Abstract:**
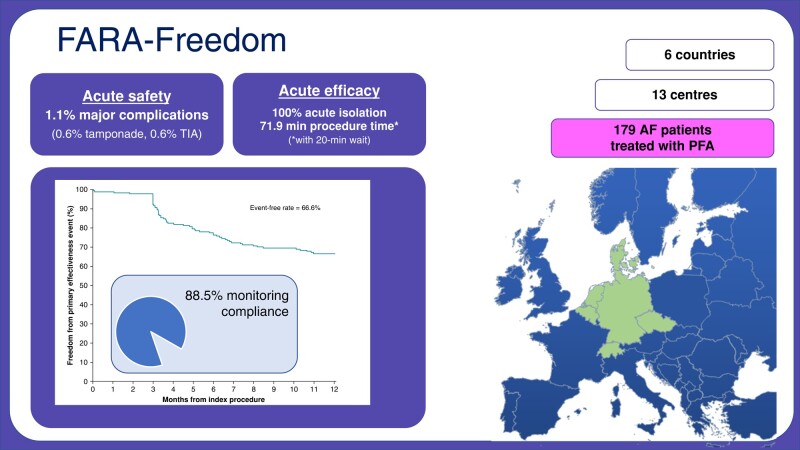

**What’s new?**

FARA-Freedom assessed the safety and effectiveness of the pentaspline PFA catheter.

- 98.9% freedom from major safety events.
- 100% acute isolation.
- 71.9- and 41.0-min procedure and left atrial dwell times, including a mandated 20-min waiting period.
- 88.5% Holter compliance at 12 months.

## Introduction

Pulmonary vein isolation (PVI) using radiofrequency ablation and cryoablation are well-established treatment approaches for paroxysmal atrial fibrillation (PAF).^1^ Pulsed-field ablation (PFA), though only recently available as a modality for preferential cardiac ablation, has already been used on thousands of patients in clinical trials and real-world registries.^2–4^ Pulsed-field ablation uses high-voltage, microsecond electrical pulses to permeabilize the cell membrane causing apoptosis where electrical fields reach sufficient strength to cause irreversible damage. Cardiac cells are more susceptible to this damage allowing for targeted ablation.

Clinical outcomes have been promising, with a favourably safety profile due to the non-thermal nature of the PFA lesion as well as comparable efficacy compared with well-established ablation techniques.^2,4–9^ Further, several large registries have demonstrated short learning curve and consistent outcomes across centres and operators of various experience.^4,7,8^

Recently, the ADVENT randomized controlled trial demonstrated non-inferiority of the pentaspline PFA system to standard-of-care thermal ablation—radiofrequency and cryoballoon ablation.^10^ However, long-term efficacy and safety data on the effectiveness of single-procedure PVI with PFA at preventing the need for antiarrhythmic drugs (AAD) usage remain limited. The aim of the FARA-Freedom Study was to evaluate long-term efficacy and safety outcome of PFA using the pentaspline catheter in patients with PAF.

## Methods

FARA-Freedom (NCT05072964) was a prospective, non-randomized, single-arm, multicentre study. The study protocol was approved by local institutional review boards at each centre. Centres were recruited based on prior PFA experience with the pentaspline catheter. The study was conducted in accordance with the Declaration of Helsinki. All patients were over 18 years old and provided written informed consent.

Thirteen centres across six countries in Europe participated in this study. Study recruitment took place between December 2021 and August 2022. Centres enrolled 3–27 patients each, and mean enrolment was of 13.2 ± 8.6 patients, with 2 sites trenching the 27-patient enrolment cap. Eligible patients were those with symptomatic PAF who previously failed AAD treatment (Classes I–IV) and were indicated for a PVI. Indication for ablation followed current guidelines and expert consensus statements. Exclusion criteria included non-paroxysmal AF, any contraindications for AF ablation, treatment with amiodarone within 3 months prior to ablation, any prior atrial ablation (accept right side cavotricuspid isthmus or for supraventricular tachycardia), prior cardiac surgery within 6 months of ablation, recent cardiovascular implantabe electronic device implant (<3 months), prior left atrial appendage closure or valve device implant, and life expectancy of <1 year.

### Pre-ablation protocol

Anticoagulation was guided by the 2017 Heart Rhythm Society Expert Consensus Statement and the 2019 American Heart Association/American College of Cardiology/Heart Rhythm Society Focused Update.^11,12^ Subjects with a CHA_2_DS_2_-VASc score ≥2 (men) or ≥3 (women) received oral anticoagulants throughout follow-up. Subjects not on anticoagulants received therapeutic anticoagulation for at least 3 weeks prior to the index procedure regardless of CHA_2_DS_2_-VASc score. Cardiac imaging using transoesophageal echocardiography or computed tomography (CT) was performed within 48 h prior to the index procedure to exclude the left atrial thrombus. Alternatively, intracardiac echocardiography (ICE) was used for this purpose intraprocedurally. All subjects without contraindications were maintained on suitable anticoagulation for at least 2 months following the index procedure.

Sedation or general anaesthesia was determined according to institutional standard of care. Femoral vein access was obtained using the Seldinger technique with ultrasound guidance recommended. A bolus of heparin was delivered prior to or immediately following transseptal puncture. Procedural activated clotting times were maintained at a minimum of 300 s using intravenous heparin bolus and/or continuous infusion.

### Pulsed-field ablation—index procedure

Pulmonary vein isolation was performed using the pentaspline PFA catheter (Farawave, Boston Scientific Inc.), deflectable sheath (Faradrive), and PFA generator (Farastar) optimized for left atrial ablation. The 12.8 F over-the-wire ablation catheter has five splines that can be deployed in the basket and flower configurations to adapt to the anatomy of the pulmonary veins. For each application, the generator delivers ultra-rapid, high-voltage electrical pulses causing irreversible electroporation of targeted cardiac tissue. The recommended procedure was that each PV receives a total of four applications in the ‘basket’ configuration with a rotation after the first two applications, followed by a second set of four applications in the ‘flower’ configuration with a rotation after the first two applications. The workflow recommended a total of eight applications per PV with additional applications allowed at the discretion of the operator. In two patients, the device was used for posterior wall isolation, and there were 20 patients with a common PV [19 left common pulmonary vein (LCPV), 1 right common pulmonary vein]; in these cases, more than the recommended eight applications per PV were applied.

Electroanatomical mapping was used at operator discretion. Oesophageal temperature monitoring or deviation was not recommended. After the last PV application, electrical isolation was confirmed following a minimum 20-min wait with the optional use of adenosine for final assessment. The status of the phrenic nerve was evaluated at the end of the index procedure. No phrenic nerve pacing was performed during ablation of the right-sided PVs.

### Endpoints

The primary safety endpoint was a composite of predefined device- or procedure-related serious adverse events with an onset within 7 days of the index procedure and PV stenosis or atrioesophageal (AE) fistula occurring at any time during the 12-month follow-up.

The primary efficacy endpoint of treatment success was defined as a composite of acute procedure success and chronic success, which included freedom from documented recurrence of AF, atrial flutter (AFL), or atrial tachycardia (AT) ≥ 30 s; use of Class I or III AAD; cardioversion after the blanking period; re-ablation for AF, AFL, or AT; or (due in part to its long half-life) the use of amiodarone at any time.

### Follow-up

Patients were followed for 12 months. Phone call assessments were completed at 7, 30, and 60 days post index procedure. At 60 days, the patient was instructed to discontinue any AADs. In-person visits were performed at 3, 6, and 12 months post index procedure with 72-h Holter ECG monitors performed at 6 and 12 months. Event monitors were used for weekly scheduled monitoring along with any symptomatic events starting after the blanking period (3 months) and continued to the end of the 12-month follow-up.

### Statistics

Continuous variables are reported as mean ± standard deviation or median (inter-quartile range). Categorical variables were summarized as count and percentage. Freedom from event survival analyses was calculated with Kaplan–Meier to determine protocol-defined endpoints and lower confidence limits (CL) and relevant event data. Odds ratios of relevant procedural characteristics and recurrence were calculated. All analysis was conducted with SAS Version 9.4 (SAS Institute Software Company). A *P* < 0.05 was considered significant.

## Results

In total, 180 patients were enrolled in the study. However, one patient was excluded from this analysis due to a persistent AF diagnosis. The remaining 179 patients with PAF underwent PVI with the pentaspline PFA catheter. The mean age was 62.3 ± 10.1 years (38.5% female) with a mean BMI of 27.3 ± 4.0 and CHADS-VASc of 1.8 ± 1.4; additional demographics are shown in *Table 1*.

**Table 1:** Patient demographics

Baseline demographics	*N* = 179
Age (years)	62.3 ± 10.1
Female	69 (38.5)
Body mass index	27.3 ± 4.0
CHADS-VASc	1.8 ± 1.4
LV ejection fraction (%)	60.9 ± 5.7 (*n* = 175)
Left atrial diameter (cm)	4.0 ± 0.5 (*n* = 175)
Comorbidities	
Dyslipidaemia	70 (39.1)
Diabetes	11 (6.1)
Hypertension	98 (54.7)
Medical history	
Cardiac ablation	7 (3.9)
Non-AF cardiac arrhythmia	27 (15.1)
Atrial flutter	20 (11.2)
Bradycardia	10 5.6)
Sick sinus syndrome	1 (0.06)
Cardiac surgery or intervention	9 (5.0)
Structural heart disease	15 (8.4)
Stroke/TIA	11 (6.1)

AF, atrial fibrillation; LV, left ventricle; TIA, transient ischaemic attack.

### Acute procedural results

Procedural characteristics are provided in *Table 2*. Procedure duration and left atrial (LA) dwell time were 71.9 ± 17.6 and 41.0 ± 13.3 min, respectively, inclusive of a protocol-mandated 20-min waiting period. The mean fluoroscopy time was 11.5 ± 7.4 min. All PVs but one right inferior PV (RIPV) were performed at 2.0 kV; the single RIPV was ablated at 1.9 kV. All PVs were acutely isolated with PFA using a mean of 9.5 applications per PV.

**Table 2:** Procedural characteristics

Procedural characteristics	*N* = 179
Procedure time (min)	71.9 ± 17.6
LA dwell time (min)	41.0 ± 13.3
Total ablation time (min)	17.8 ± 10.1
Fluoroscopy time (min, *n* = 178)	11.5 ± 7.4
Transoesophageal echo	67% (120)
Intracardiac echo	29.6% (53)
Acute vein isolation	100% (702/702 PVs)
First pass vein isolation	98.6% (692/702 PVs)
Applications per PV	9.5 ± 3.0
CTI ablation performed	14/179 (7.8%)
CTI-documented bidirectional block	13/14 (92.9%)
CTI-duration of CTI ablation (min)	17.0 ± 17.4

CTI, cavotricuspid isthmus; PV, pulmonary vein.

### Rhythm monitoring compliance

Rhythm monitoring compliance was notably high during follow-up. Patients were given event monitors to record weekly EKGs and as needed for symptoms starting after the 3-month blanking period. Event monitor compliance was 88.4% for the scheduled weekly transmissions. Additional rhythm monitoring compliance details can be found in *Table 3*. For the 72-h Holter monitor, compliance was 92.1 and 88.5% at 6- and 12-month follow-up, respectively.

**Table 3:** Rhythm monitoring compliance

Rhythm monitoring compliance	*N* = 179
**Event monitor (EM) weekly compliance**	
Number of scheduled EM records	5184
Number of unscheduled EM records	9217
Number of EM records per subject	81.4
Mean EM weekly compliance	88.4% (5184/5866)
**Holter monitor compliance**	
Holter Monitor at 6 months	92.1% (163/177)
Holter Monitor at 12 months	88.5% (154/174)

### Safety

**Figure 1.**
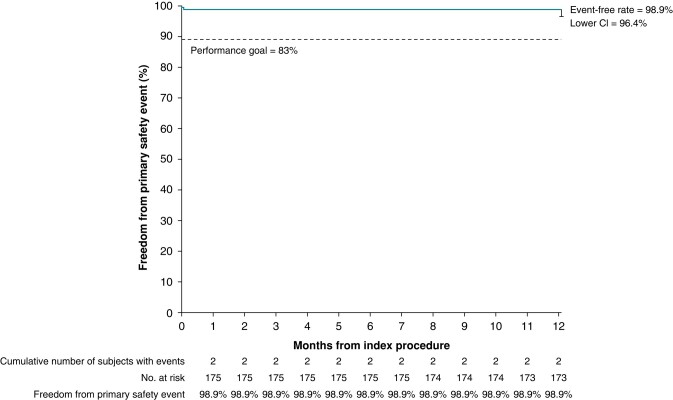
Safety. CL, confidence limits.

The composite safety endpoint occurred in two patients (1.1%; *Figure 1*), one cardiac tamponade and one transient ischaemic attack (TIA). The tamponade was suspected to be caused by LA perforation due to the guidewire and was stabilized during the procedure. The TIA occurred 2 days after the ablation procedure in a patient with a clotting disorder. Imaging studies were performed with no abnormalities observed. The TIA resolved without further sequelae. There were no instances of clinically apparent coronary spasm, PV stenosis, or AE fistula. Phrenic nerve function was assessed during the index procedure. There were four instances of transient phrenic nerve palsy, but all cases had documented resolution during the procedure.

### Efficacy

**Figure 2.**
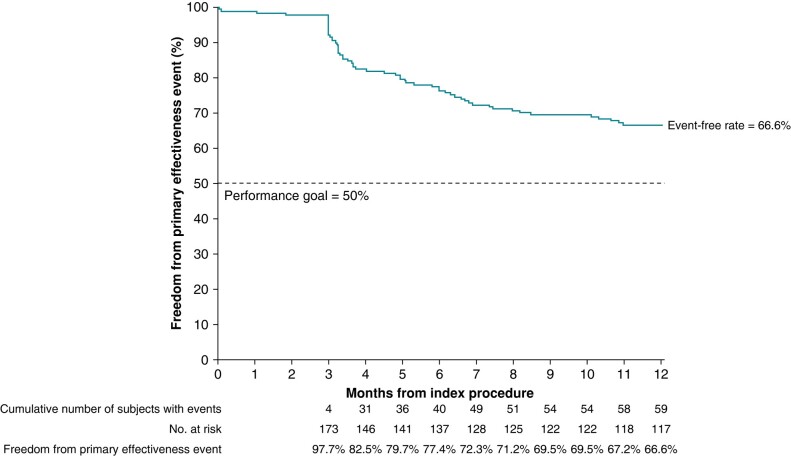
Effectiveness.

At 12-month follow-up, freedom from composite primary effectiveness endpoint was 66.6% (*Figure 2*). The primary failure modes are shown in *Table 4*. The most common mode of primary treatment failure was recurrent atrial tachyarrhythmia, with AF being the most common (31 patients) followed by AT (7 patients). Twelve patients had failure due to Class I/III AADs being used after the blanking period (Day 90). Of those 12 subjects, 5 discontinued their AAD medication between Days 92 and 95.

**Table 4:** Primary efficacy failure mode

First primary effectiveness failure mode	
Any primary effectiveness failure mode	59
Acute procedural failure	0
PVI performed with non-PFA device	0
Post-blanking detectable AF/AFL/AT/SVT	41
Post-blanking detectable AF	31
Post-blanking detectable AFL	0
Post-blanking detectable AT	7
Post-blanking detectable SVT	3
Post-blanking cardioversion for AF/AFL/AT	0
Post-blanking use of type I/III AAD	12
Any re-ablation for AF/AFL/AT	3
Any non-procedural use of amiodarone	3

AFL, atrial flutter; AT, atrial tachycardia; SVT, supraventricular tachycardia.

Early recurrence of AF (ERAF), defined as recurrence of AF during the 3-month blanking period, occurred in 14 patients (8.1%), 9 of which also had recurrent AF during the post-blanking period. Thirteen of these 14 patients met the definition of treatment failure (7 for arrhythmia recurrence, 3 for AAD, 1 for re-ablation, and 2 for amiodarone).

Patient quality of life scores were captured at baseline and at 12-month follow-up using the AFEQT and EQ-5D-3L, which both significantly improved with an average increase of 23.8 and 6.4 in the scores, respectively.

### Repeat ablations

Out of 179 patients, 11 (6.1%) returned for repeat ablation at a mean follow-up of 7.3 ± 2.7 months. One patient had a repeat ablation performed during the blanking period, with the remaining 10 patients having repeat ablation post-blanking. These 11 patients had a mean age of 64 years, a BMI of 28.9 ± 3.9, and a CHADS-VASc score of 2.4 ± 1.2. At repeat ablation, 65% (26/40 PVs) of the PVs were durably isolated, and 2 patients (18.2%) had all treated PVs durably isolated. Eight patients out of these 11 patients (72.8%) needed ablation beyond the PVs, including 5 LA roofs, 2 LA floors, 2 mitral isthmuses, 2 posterior walls, and 1 focal in LA. The most common reconnection was the RSPV (6/11 PVs; *Table 5*). Interestingly, there were significantly more repeat ablations in patients with a LCPV. Of 19 patients with LCPVs included in the study, 5 (26.3%) returned for repeat ablation vs. 6 in the remaining patient cohort (3.8%; *P* = 0.002), although 3/5 LCPVs (60%) remained durably isolated. There was no PV stenosis in repeat ablation patients.

**Table 5:** Repeat ablation

Parameter	% (*n*/*N*)
Recurrent AF	45.5% (5/11)
Recurrent CTI-mediated AFL	0.0% (0/11)
Atypical flutter (LA)	18.2% (2/11)
Atypical atrial flutter (RA)	0.0% (0/11)
Atypical flutter (bi-atrial)	0.0% (0/11)
Atrial tachycardia (RA)	0.0% (0/11)
Atrial tachycardia (LA)	18.2% (2/11)
AVRT/accessory pathway	0.0% (0/11)
AVNRT/slow pathway modification	0.0% (0/11)
PVCs	0.0% (0/11)
Ventricular tachycardia	0.0% (0/11)

Some subjects had multiple ablation indications. AVRT, atrioventricular reentrant tachycardia; AVNRT, atrioventricular nodal reentry tachycardia; PVCs, premature ventricular contractions.

## Discussion

The FARA-Freedom Study provides long-term, single-procedure outcomes in PAF patients treated with the pentaspline PFA catheter. Despite a rigourous definition of treatment success, e.g. not allowing membrane-active AADs or re-ablation, 12-month efficacy was comparable to thermal ablation outcomes and other PFA technologies. In this study, there was high rhythm monitoring compliance contributing to the rigour of the clinical assessment. These results also demonstrate an excellent safety profile for patients undergoing PVI using this PFA technology.

### Safety

Pulsed-field ablation continues to demonstrate favourable safety outcomes, eliminating the thermal complications seen with radiofrequency and cryoballoon ablation, such as phrenic nerve paralysis, PV stenosis, and AE fistula. In this study, there were only two reported composite safety events, one of which was a TIA in a patient with a clotting disorder and predisposition for embolic complications. In good agreement with reports of near zero risk of long-term phrenic nerve damage,^4,6,7^ here, there were only intraprocedural phrenic nerve impairments, all of which resolved during the procedure. Additionally, it is notable that though there is recent interest in coronary artery spasms as a result of vascular muscle stimulation by the PFA electrical field,^13^ this study saw no instances of clinically manifest coronary spasm. Pulmonary vein stenosis continues to be a rare event regardless of ablation modality, but a recent publication on the ADVENT RCT reports the subclinical PV narrowing in patients with available imaging data.^14^ While no clinical PV stenosis occurred, they found that more PV narrowing is more likely to be present following thermal ablation.^14^ Similarly, the present study had no incidents of PV stenosis. There are recent reports of acute kidney damage associated with extensive ablation sets.^15^ While a recent study demonstrates that these effects can be completely prevented by post-ablation hydration,^16^ the present study saw no acute kidney damage, though it should be noted that this study used ablation sets for PVI alone, which did not approach the high numbers associated with kidney damage,^16^ though subclinical effects of minor haemolysis may have gone undetected. Overall, there were no complications leading to permanent sequelae in any patient.

### Efficacy

The present study had an overall 66.6% composite effectiveness, driven by recurrence of AF, I/III AAD usage, re-ablation, and amiodarone usage. This rate is similar to reports on other PFA devices, where Verma *et al.*^17^ reported a 66.2% effectiveness in the PAF patients in the PULSED-AF study and Duytschaever *et al.*^18^ reported a 70.9% rate in the INSPIRE study. Though it is notable that these studies allowed AAD usage, they may have apparent ‘higher’ effectiveness than studies like the present, where usage of AADs post-blanking was defined as treatment failure. The present findings are also comparable to recent studies with the pentaspline catheter; for instance, the MANIFEST and EUPORIA registries reported effectiveness of 73 and 78%, respectively,^4,7^ and the ADVENT randomized clinical trial reported an overall effectiveness of 73%.^2^ It should be noted that the large registries MANIFEST and EUPORIA do not have the stringent endpoint and follow-up criteria of the present study.^4,7^ Efficacy is also comparable to legacy data from radiofrequency and cryoablation catheters, where efficacy ranges from 64 to 75%,^19,20^ in line with recent data from ADVENT where thermal ablation was 71% effective.^2^ Additionally, repeat ablation in this study is similar to a recent report on mapping data from 25 of 360 patients that returned for re-ablation following PVI with the pentaspline catheter.^21^ Tohoku *et al.*^21^ reported that PV reconnection was low in these patients and that the most common reason for re-ablation was macro-reentrant AT. The reported AT recurrence rate (4.4%, 16/325) is similar to the present findings (3.9%, 7/179). It seems likely that continued workflow optimization with this still novel pentaspline catheter is likely to further reduce AT recurrence.

The range of reported effectiveness from different studies may result from multiple factors, such as patient compliance, monitoring strategies, and endpoint definitions. Across studies, there is a relationship between patient compliance (i.e. rates of rhythm monitoring) and effectiveness (per protocol, or documented recurrence). When compliance is high, effectiveness can appear lower. For instance, historical RF trials DIAMOND AF and SMART AF had intersecting compliance and effectiveness rates.^22,23^ DIAMOND AF had low compliance (61%) and high effectiveness (79%),^22^ while SMART AF had high compliance (84%) and relatively lower effectiveness (72.5%, documented recurrence).^23^ Thus, the present ∼90% compliance rate should be factored into interpretation of endpoint data. Differences in trial design, conduct, and monitoring protocols can also make comparing outcomes challenging. From the studies published to date, it is clear that with more consistent and rigourous monitoring, more arrhythmia recurrences will be captured and reflected as reduction in long-term effectiveness. For instance, it should be expected that more rigourous transtelephonic monitoring (TTM, such as weekly vs. monthly) would detect more asymptomatic AF episodes resulting in a higher documented recurrence rate.^24^ Similarly, length of Holter monitoring may be expected to drive outcomes, with a longer monitoring period (the present 72 h, for instance) detecting more recurrence than the standard 24-h Holter monitoring. Further discrepancies arise in defining what should be a clinically meaningful endpoint for effectiveness. Trials often use the first recurrent 30-s atrial tachyarrhythmia episode as treatment failure.^10,19,20^ However, recent data suggest that in some treatment populations, there may be a ‘peak’ in recurrence post-blanking that does not reflect long-term effectiveness.^25^ It is also important to emphasize that, even though some operators may still be in their learning curve with PFA, the novel pentaspline catheter achieves similar efficacy to well-established thermal technologies that have been used for many years.^10^

### Limitations

FARA-Freedom was designed as a post market clinical follow-up study assessing single-procedure success based on standard of care in 6 countries and at 13 centres. This was a single-arm study with 12-month follow-up. The patient rhythm monitoring compliance was notably high in this study, this combined with 72-h Holter monitor, and strict treatment success definitions make direct comparisons to other study outcomes challenging.

## Conclusions

In this prospective, non-randomized, multicentre study, PVI using the pentaspline PFA catheter was effective in treating PAF patients despite rigourous endpoint definitions and high monitoring compliance and demonstrated favourable safety.

## Data Availability

The data from this clinical trial may be made available to other researchers in accordance with Boston Scientific’s Data Sharing Policy (http://www.bostonscientific.com/en-US/data-sharing-requests.html).
